# Effect of exercise on cluneal nerve entrapment neuropathy: a case report

**DOI:** 10.1186/s13256-024-04641-w

**Published:** 2024-07-25

**Authors:** Burcu Özüberk, Mine Argalı Deniz, Feray Cinevre Soyupek

**Affiliations:** 1 Faculty of Health Sciences, Department of Physiotherapy and Rehabilitation, Kırklareli University, Kırklareli, Turkey; 2 Department of Physiotherapy and Rehabilitation, Suleyman Demirel University, Research and Application Hospital, Isparta, Turkey; 3 Faculty of Medicine, Department of Physical Medicine and Rehabilitation, Suleyman Demirel University, Isparta, Turkey

**Keywords:** Nn. clunium superiores, Entrapment, Low back pain, Exercise

## Abstract

**Background:**

Low back pain is an important disability problem frequently encountered in the clinic. In the literature, it has been shown that neuropathic pain in chronic low back pain is quite common in patients. Although superior cluneal nerve entrapment syndrome is an underdiagnosed cause of low back and leg pain, differential diagnosis is very important anatomically and clinically. The superior cluneal nerves are pure sensory nerves that innervate the skin of the upper part of the buttocks. In the literature, methods such as surgery, nerve blockade, prolotherapy, and acupuncture have been used in the treatment of cluneal nerve entrapment syndrome, but there are no studies on exercise. In this case report, our aim is to explain the importance of differential diagnosis in cluneal nerve entrapment syndrome, which is one of the common causes of low back pain in the clinic, and the effects of exercise in this disease.

**Case presentation:**

A 22-year-old, Turkish-ethnicity, male patient with complaints of low back pain, neck–back pain, and weakness did not use alcohol or cigarettes. In his family history, there was a history of diabetes in the mother and diabetes and heart failure in the father. He had a history of osteoporosis, epilepsy, asthma, sarcoidosis, and cardiac arrhythmia. The patient reported that he suffered from constipation three to four times a month. As a result of the detailed evaluation, the planned exercise prescription was taught to the patient, and after it was confirmed that the patient did the exercises correctly for 3 days, the exercise brochure was given and followed as a home exercise program for 8 weeks.

**Conclusions:**

Lumbar stabilization exercises, gluteal muscle strengthening exercises, thoracolumbar fascia mobilization, and stretching exercises, which will be given in accordance with the clinical anatomy of the disease after the correct diagnosis in cluneal nerve entrapment syndrome, have been beneficial for the patient. However, we think that randomized controlled studies with a large sample will contribute to the literature.

## Background

Low back pain is an important health problem that can affect the functional activity level of individuals [1]. Epidemiological studies have explained that low back pain, which can occur with different pathologies, may also be of neuropathic origin [2]. Cluneal nerve entrapment syndrome (CNES) that can cause low back pain was first reported by Strong and Davila in 1957 [3]. The superior cluneal nerves are sensory nerves known to originate from the posterior rami of the lower thoracic and lumbar nerve roots. Its course in the tissue starts from the superomedial and extends to the inferolateral and ends at the hip [4]. The nerve can be trapped at any point where it penetrates the thoracolumbar fascia near the iliac crest, or it can be trapped spontaneously as it passes through the osteofibrous tunnel formed by the superior iliac crest and the thoracolumbar fascia [5].

In low back pain due to CNES, the patient’s pain is exacerbated by lumbar movements such as extension, flexion, rotation, long standing, sitting, walking, and rolling [6]. It may also mimic radiculopathy due to leg pain and may cause intermittent claudication due to low back pain worsened by prolonged walking [7, 8]. In CNES, the detection of a Tinel-like mark at the thoracolumbar fascia penetration point and the disappearance of pain upon nerve blockade are diagnostically important [9, 10]. In the literature, the results of treatments such as surgical techniques, nerve blockade, prolotherapy, and acupuncture have been mentioned in the treatment of CNES, but there are no studies on exercise [6].

The aim of this case report is to explain the importance of differential diagnosis in CNES, which is one of the common causes of low back pain in the clinic, and the effects of exercise in this disease.

## Case presentation

A 22-year-old, Turkish-ethnicity, male patient with complaints of low back pain, neck–back pain, and weakness presented to the Physical Therapy and Rehabilitation Polyclinic of Süleyman Demirel University Research and Application Hospital in August 2021 due to increasing pain. After examining the results of magnetic resonance imaging and than nerve block together with a detailed physical examination by physiatrist, he was referred to a physiotherapist for exercise practices for CNES.

Our patient, who was single and did not work anywhere, did not use alcohol or cigarettes. His mother had diabetes, and his father had diabetes and heart problems. He had a history of osteoporosis, epilepsy, asthma, sarcoidosis, and cardiac arrhythmia. The patient reported that he suffered from constipation three to four times a month. In addition, the patient was using inhaled medication, alendronat (one per week), aripiprazol (20 mg), paroxetine (40 mg), lamotrigine (100 mg, morning/evening), dexletroprofen (one to two per week, when in pain).

Our patient, whose consent was taken for the study, had good exercise compliance and did not encounter any unexpected adverse events during the treatment.

Body mass index was 19.5 kg/m^2^. Tinel’s sign at the point where the superior cluneal nerve pierces the thoracolumbar fascia, Kemp’s test, piriformis test, straight leg raising test, and posterior friction test were positive. There was increased pain on palpation in the L4–5 vertebrae and iliac crest. There was also a decrease in patellar and Achilles reflexes. There was no problem in light touch, sharp–blunt, and hot–cold sensory evaluations. It was determined that the pain was aggravated with lumbar flexion and spread to the lower extremity. Lumbar pelvic tilt was 15°, and pelvis was anterior tilt. Muscle shortness was detected in the latissimus dorsi, pectoralis major, pectoralis minor, hamstring, and lumbar extensor muscles. There was functional lumbosacral scoliosis with curves toward the left.

In the evaluation of the patient’s lumbar region joint range of motion, flexion was in full range but painful, and there was limitation in extension, right/left lateral flexion, and rotation. Abdominal, back extensor, and gluteal muscle strength were decreased. In our patient who was included in the exercise program, goniometric measurements of joint range of motion and muscle strength analysis were made in detail before and after treatment (Table 1).

**Table 1:** Results of normal range of motion evaluation and muscle test before and after treatment

	Before treatment	After treatment
Lumbar region joint range of motion test		
Flexion	90°	90°
Extension	5°	15°
Lateral flexion (right/left)	15°/15°	25°/25°
Rotation (right/left)		
Muscle test		
Rectus abdominis	4	5
Lower abdominals	4	4
Oblique trunk flexors (right/left)	3/3	4/4
Back extension	3	5
Hip flexors (right/left)	4/4	5/5
Hip extensor (right/left)	4/4	5/5
Hip abductors (right/left)	3/3	4/4

The patient’s pain around the right iliac crest and back was 8 according to visual analog scale (VAS), which was rhythmic, periodic, intermittent aching, throbbing, and fatigued. Pain worsened with prolonged sitting, trunk flexion, and heavy lifting. New York Posture Rating Test was used to evaluate the patient's posture analysis, Oswestry Low Back Pain Disability Questionnaire and Roland Morris Disability Questionnaire were used to assess functional status, short form SF-36 was used for quality-of-life assessment, and Beck Depression Questionnaire was used to assess mood (Table 2).

**Table 2:** Results of evaluation parameters before and after treatment

Evaluation parameters	Before treatment	After treatment
New York posture rating	39	41
Oswestry Low Back Pain Disability Questionnaire	12	6
Roland Morris Disability Questionnaire	9	11
Short form SF-36	85	78
Beck Depression Questionnaire	23	13

As a result of the detailed evaluation, the planned exercise prescription was taught to the patient, and after it was confirmed that the patient did the exercises correctly for 3 days, the exercise brochure was given and followed as a home exercise program for 8 weeks.

**Fig. 1:**
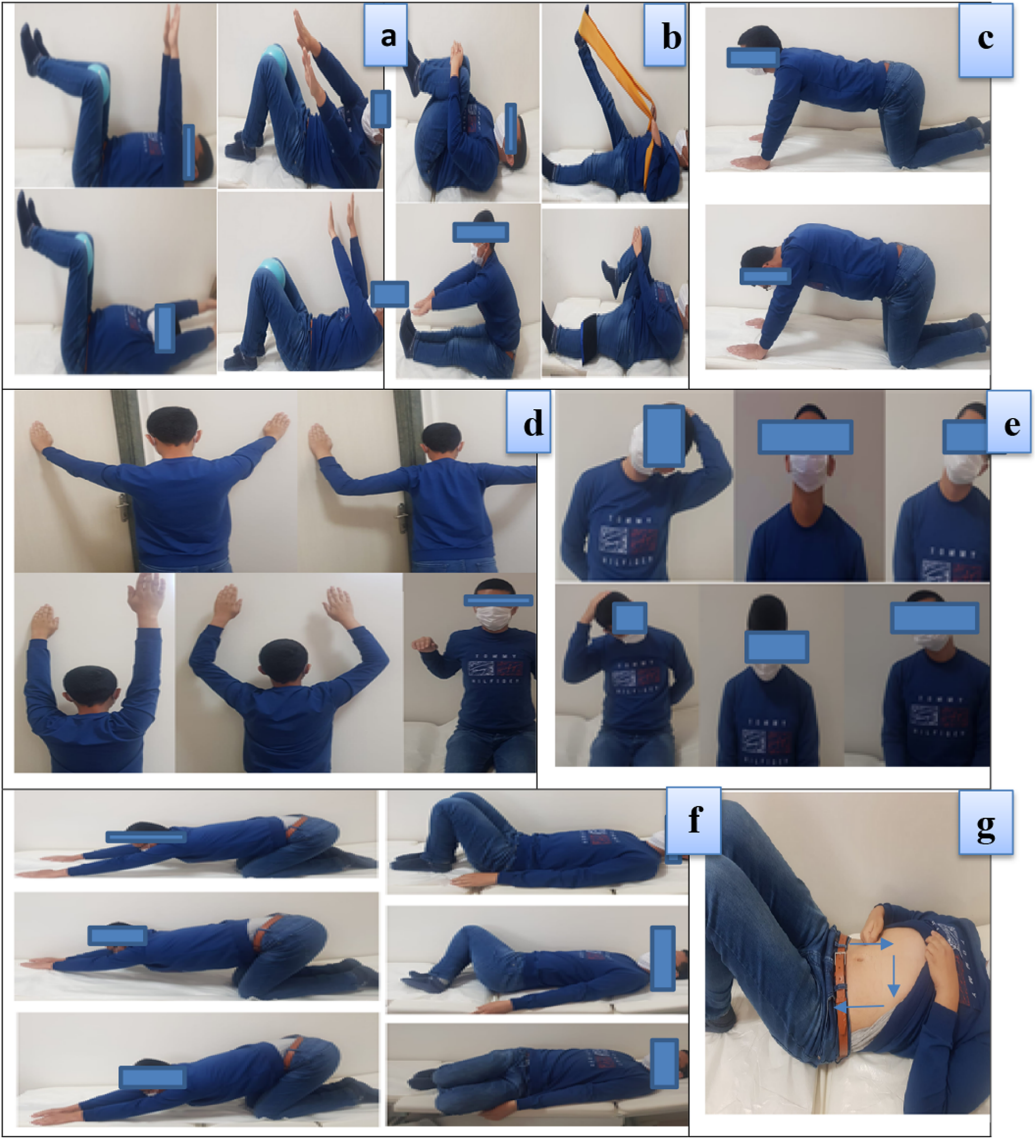
Exercise program. **a** Abdominal strengthening exercises. **b** Hamstring/iliopsoas/lumbar extensors stretching exercises. **c** Mobilization exercise. **d** Posture exercises. **e** Neck joint range of motion and stretching exercises. **f** Thoracolumbar fascia stretching exercises. **g** Colon massage: Abdominal/intestinal massage can also be prescribed instead of colon massage. Abdominal massage for intestinal health: If you rub your abdomen with circular movements starting from the lower right part of your abdomen to the upper right, then to the upper left and then to the lower left, you can relieve intestinal laziness caused by inactivity to some extent (Blue arrows)

The exercise program included abdominal–gluteal region strengthening exercises, core stabilization exercises, posture exercises, hamstring/iliopsoas/lumbar extensor muscles stretching exercises, thoracolumbar fascia stretching/mobilization exercise, and colon massage (Fig. 1).

## Discussion and conclusions

In this case report, a decrease in pain around the iliac crest and back was observed in our patient, whom we followed up with a home exercise program. In addition, improvements were noted in joint range of motion, posture, muscle strength, functional status, quality of life, and mental status assessment.

CNES is often confused with facet syndrome, lumbar disc problems, or iliolumbar syndrome because its clinical features are similar. Tenderness on the iliac crest and the trigger point 7 cm lateral to the processus spinosus are important for diagnosis [11]. The treatment was effective with the correct diagnosis made with similar findings in our patient.

Speed *et al*. [12] reported that overactivation of the thoracolumbar erector spina and latissimus dorsi muscles would increase lumbar region pain, and thus the superior cluneal nerve might be susceptible to entrapment. Erdem *et al*. [11] reported that posture is affected in the neuropathy of the superior cluneal nerve due to the lower crossed syndrome, described by Janda in 1979, and the core arrangement in the spine will deteriorate. In the light of these studies, we noted that, thanks to the core and posture exercises we applied to our patient, there was an increase in muscle strength and posture improved from moderate to good in the New York posture evaluation. In addition, we have seen that lumbar stabilization exercises, gluteal muscle strengthening exercises, and stretching and mobilization exercises of the thoracolumbar fascia are important in the treatment.

Konno *et al*. [2] demonstrated a mechanism that causes pseudo-sciatic leg symptoms of entrapment block in the transition from the osteofibrous tunnel in the fascia covering the iliac crest of L4–5 branches. We encountered pseudo-sciatic leg symptoms in our patient, who had complaints of shortness in the hamstring muscle group and along the sciatic nerve tracing, and a decrease in pain was noted with stretching exercises in the hamstring muscle group.

Morimato [13] and Kuniya [6] reported that, while CNES complaints aggravate owing to dynamic compression to the lumbar region during long-term standing, sitting, and walking, and because the nerve is stretched in trunk flexion, the complaints are relieved in trunk flexion and hip extension to the same side. In our patient, we observed that complaints increased with lumbar flexion and antalgic posture developed due to pain. In this context, the patient’s functional status, quality of life, and mental status improved by being supported with patient training and posture exercises.

Our patient said that, after the treatment, his pain decreased while leaning forward and he was able to walk longer on the road.

The inclusion of breathing exercises in the rehabilitation program can be considered as a limitation of our study. Asthma is a condition that increases respiratory workload and affects the functional levels and comfort of patients in daily life.

As a result, lumbar stabilization exercises, gluteal muscle strengthening exercises, thoracolumbar facia mobilization, and stretching exercises, which will be given in accordance with the clinical anatomy of the disease after the correct diagnosis in CNES, have been beneficial for the patient. However, we think that randomized controlled studies with a large sample will contribute to the literature.

## Data Availability

Not applicable.
